# Networks of Causal Linkage Between Eigenmodes Characterize Behavioral Dynamics of Caenorhabditis elegans

**DOI:** 10.1371/journal.pcbi.1009329

**Published:** 2021-09-10

**Authors:** Erik Saberski, Antonia K. Bock, Rachel Goodridge, Vitul Agarwal, Tom Lorimer, Scott A. Rifkin, George Sugihara

**Affiliations:** 1 Scripps Institution of Oceanography, University of California, San Diego, La Jolla, California, United States of America; 2 Division of Biological Sciences, University of California, San Diego, La Jolla, California, United States of America; Vrije Universiteit Amsterdam, NETHERLANDS

## Abstract

Behavioral phenotyping of model organisms has played an important role in unravelling the complexities of animal behavior. Techniques for classifying behavior often rely on easily identified changes in posture and motion. However, such approaches are likely to miss complex behaviors that cannot be readily distinguished by eye (e.g., behaviors produced by high dimensional dynamics). To explore this issue, we focus on the model organism *Caenorhabditis elegans*, where behaviors have been extensively recorded and classified. Using a dynamical systems lens, we identify high dimensional, nonlinear causal relationships between four basic shapes that describe worm motion (eigenmodes, also called “eigenworms”). We find relationships between all pairs of eigenmodes, but the timescales of the interactions vary between pairs and across individuals. Using these varying timescales, we create “interaction profiles” to represent an individual’s behavioral dynamics. As desired, these profiles are able to distinguish well-known behavioral states: i.e., the profiles for foraging individuals are distinct from those of individuals exhibiting an escape response. More importantly, we find that interaction profiles can distinguish high dimensional behaviors among divergent mutant strains that were previously classified as phenotypically similar. Specifically, we find it is able to detect phenotypic behavioral differences not previously identified in strains related to dysfunction of hermaphrodite-specific neurons.

## Author summary

A primary reason for studying *C. elegans* is the possibility of achieving an end-to-end understanding of animal behaviour—one that mechanistically links genes to behaviour through neurobiology. With the genome and connectome fully mapped, techniques for measuring the behavior of *C. elegans* have traditionally relied on easily identified changes in posture and motion. Such approaches, however, can miss complex and more subtle behaviors that cannot be readily distinguished by eye. To explore this issue, we develop an attractor-based network approach to show that the four basic shapes classically used to describe worm position can be related to each other in a network that represents how the shapes interact with each other over time. That is, we study not just worm poses, but how the poses interact. Importantly, we find that these interaction networks can distinguish subtle high dimensional behaviors among mutant strains previously classified as phenotypically similar, thereby making genetic distinctions not previously possible. Thus, the value of this attractor-based network approach is its ability to detect subtle differences that can discriminate macroscopic behavior at the gene-mutation level. We anticipate these methods will be of interest to behaviorists, neuroscientists, and geneticists studying the connection between behavior and genes.

## Introduction

*Caenorhabditis elegans* has long served as an important model species for the comprehensive study of behavioral dynamics from genes to connectome to behavior. Sydney Brenner sought to uncover relationships between *C. elegans* genetics and behavior nearly five decades ago [1], and White et al. mapped the entire neural connectome of the organism almost four decades ago [2]. Despite being a relatively neurologically simple organism, *C. elegans* exhibits complex, chaotic dynamics [3], which has made characterizing the full detail of its behavior a difficult task.

To gain a handle on the dynamics of complex systems, we generally seek to capture them in a model that describes the relationships between relevant variables. These relationships, however, are often not known, and so a natural first step is to identify and measure the (dynamic) causal influences among variables using observational data.

In nonlinear systems, the strengths of dynamic influences typically vary as a function of the time delay, or *lag*, between cause and effect. Thus, it is prudent to measure dynamics causal influence not for a single time lag, but across a suite of time lags [4,5]. This suite of causal interaction strengths–a causal interaction *profile*–provides a fingerprint of the underlying dynamics. It might therefore also be used to quantitatively characterize those dynamics (Box 1). Here we test this concept by describing *C. elegans* behavior in terms of the causal interaction profiles between its relevant variables of motion.

**Box 1**

**Convergent Cross Mapping (CCM) identifies nonlinear relationships between timeseries variables by measuring how one timeseries “maps” onto another. If mapping time series Y onto time series X can be used to predict previous values of X, then X has a causal influence on Y**.

**Take, for example, the coupled logistic equations**:

$$x_{t+1}=x_{t}(3.8-3.8x_{t})$$

$$y_{t+1}=y_{t}(3.5-3.5y_{t}+0.1x_{t-4})$$

**In this system, values of *x* have an influence on future values of *y*, but *y* has no influence on *x*. CCM tells us that we should be able to use values of Y to accurately map onto (predict) values of X. However, without knowing the underlying equations, the delay of the interaction is not immediately clear. To empirically identify the delay in this coupling, we perform CCM with various target delays (e.g., use y_t+1_ to predict values of x_t_, x_t-1_, x_t-2_ etc.) From the equations, we know y_t+1_ should most accurately map onto x_t-4_. The profile of resolved CCM strength versus target delay is shown in the far left panel. As expected, the strongest coupling is found with a target delay of 4 timesteps (red line)**.

**Fig 1:**
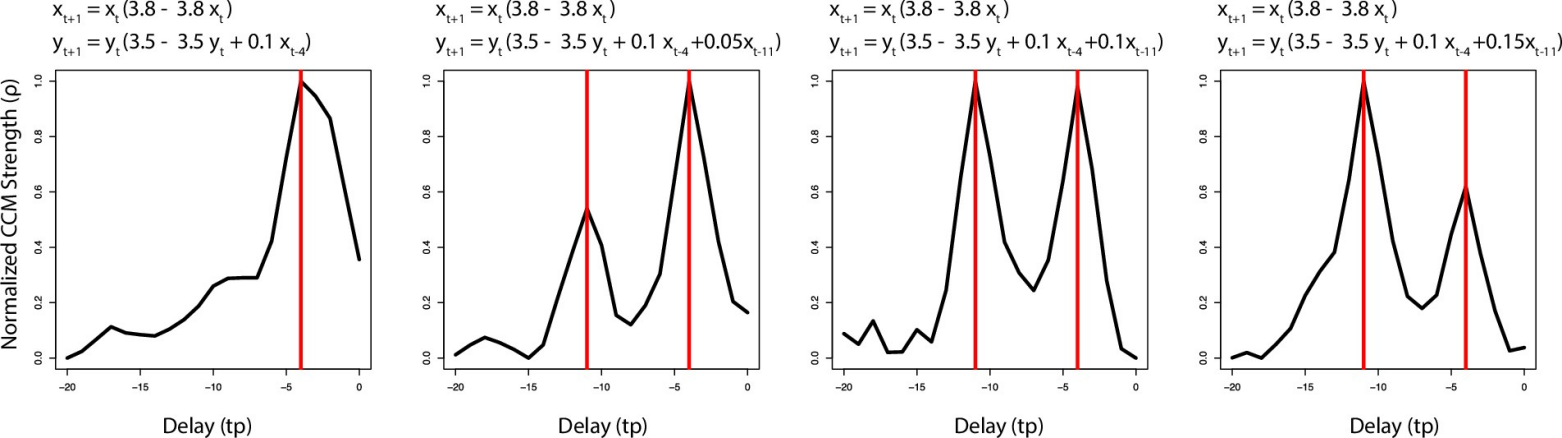
Interaction profiles of the influence of X on Y for four coupled logistic models. The red vertical lines represent the delays in the coupling determined by the equations. Note that peaks in causal influence (normalized CCM strength) align with the coupling delays determined by the equations.

**In dynamical systems, it is typical for coupling to occur on multiple timescales. We can simulate this by including an additional coupling term in the function for y_t+1_ (plots 2–4). However, this coupling occurs with a delay of 11 timesteps. With this coupling, the profile identifies two peaks: one at 4 timesteps, another at 11. As the coupling strength of the delay 11 is increased, the relative mapping between the two delays measured through CCM changes (Fig 1)**.

**In highly complex systems, these profiles are likely a synthesis of many interactions, both direct and indirect, representing an average delay in information transfer from one variable to another**.

Through a series of seminal works, it has been established that the relevant variables of *C. elegans* motion on a surface are the leading eigenvectors of worm body pose, colloquially referred to as “eigenworms” [6–8]. Therefore, we ask here whether profiles of causal interaction between these relevant variables (eigenworms) may be able to provide a rich quantitative characterization of worm motion. For this proof of concept, we use Convergent Cross Mapping (CCM) as a measure of causal interaction [9] though this general approach might be applied with other methods in future work (e.g., [5]). CCM detects weak nonlinear interactions such as might be expected between orthogonal linear modes of body pose displaying nonlinear dynamics.

Previous studies have used a variety of methods to extract phenotypic features or behavioral motifs to cluster mutant strains, such as biomechanical profiling (e.g. [10]), construction of dictionaries of features (e.g. [7,11,12]) empirical mode decomposition of body curvature of the worm (e.g. [13]), and machine learning [14]. Because nonparametric models have successfully classified *C. elegans* behaviors [3], we expect methods specifically designed to identify relationships in nonlinear systems (e.g. [4,9]) to be well suited to explore differences between behaviors as well.

We find that the set of causal interaction profiles between all ordered pairs of the first four eigenworms provides a robust and useful quantitative characterization of worm motion behavior.

## Results

**Fig 2:**
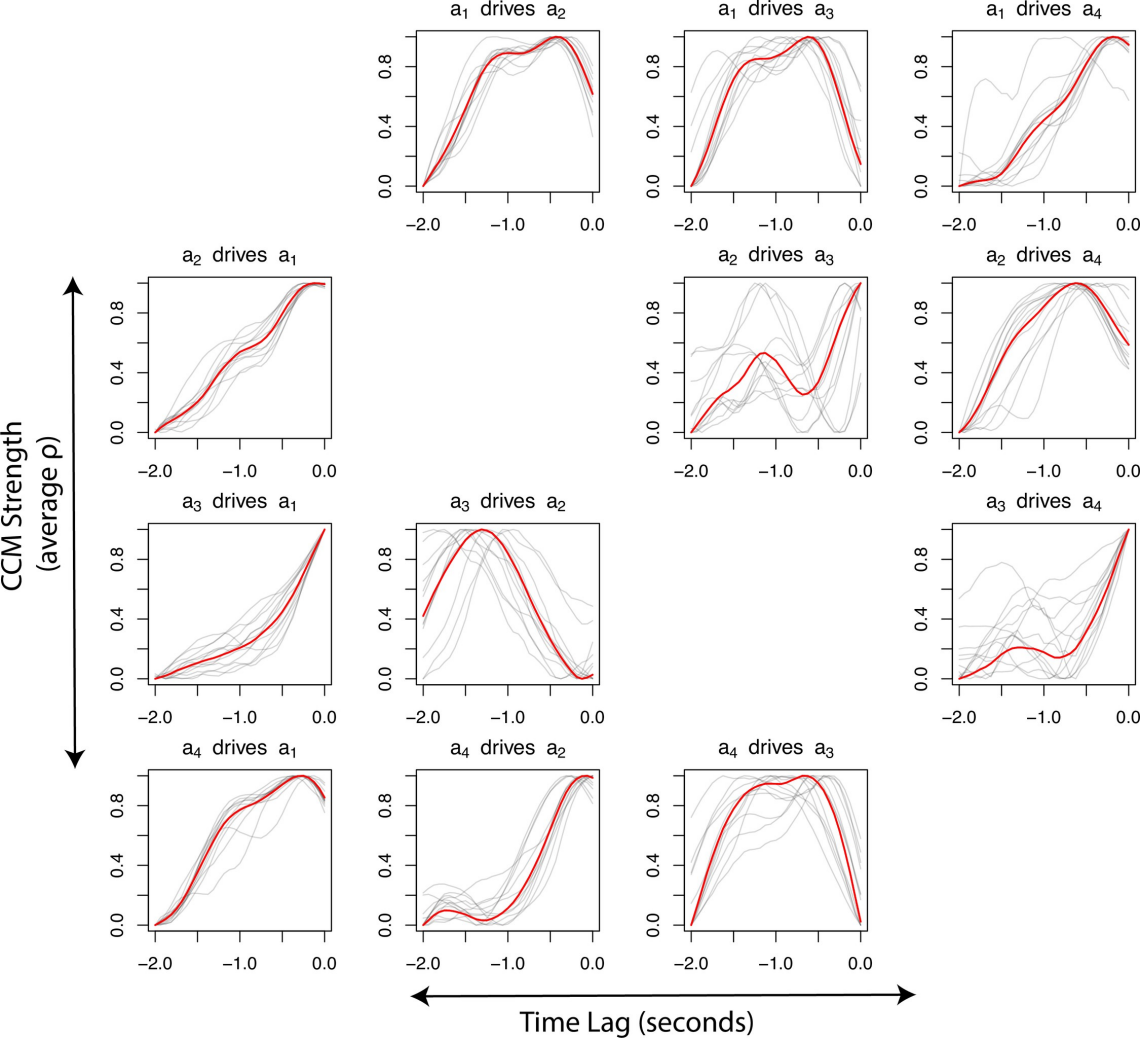
Interaction profiles represented by time-lag versus normalized CCM correlation coefficient (ρ) for each pair of the first four eigenmodes for 12 foraging worms (grey lines—individuals, red line—average). Note that pairs of eigenmodes interact at different timescales, however, these relationships are relatively consistent across individuals. The profiles here are normalized to account for variation in CCM magnitude across timeseries (see S1 Fig). The non-normalized profiles can be found in S1 Fig.

**Fig 3:**
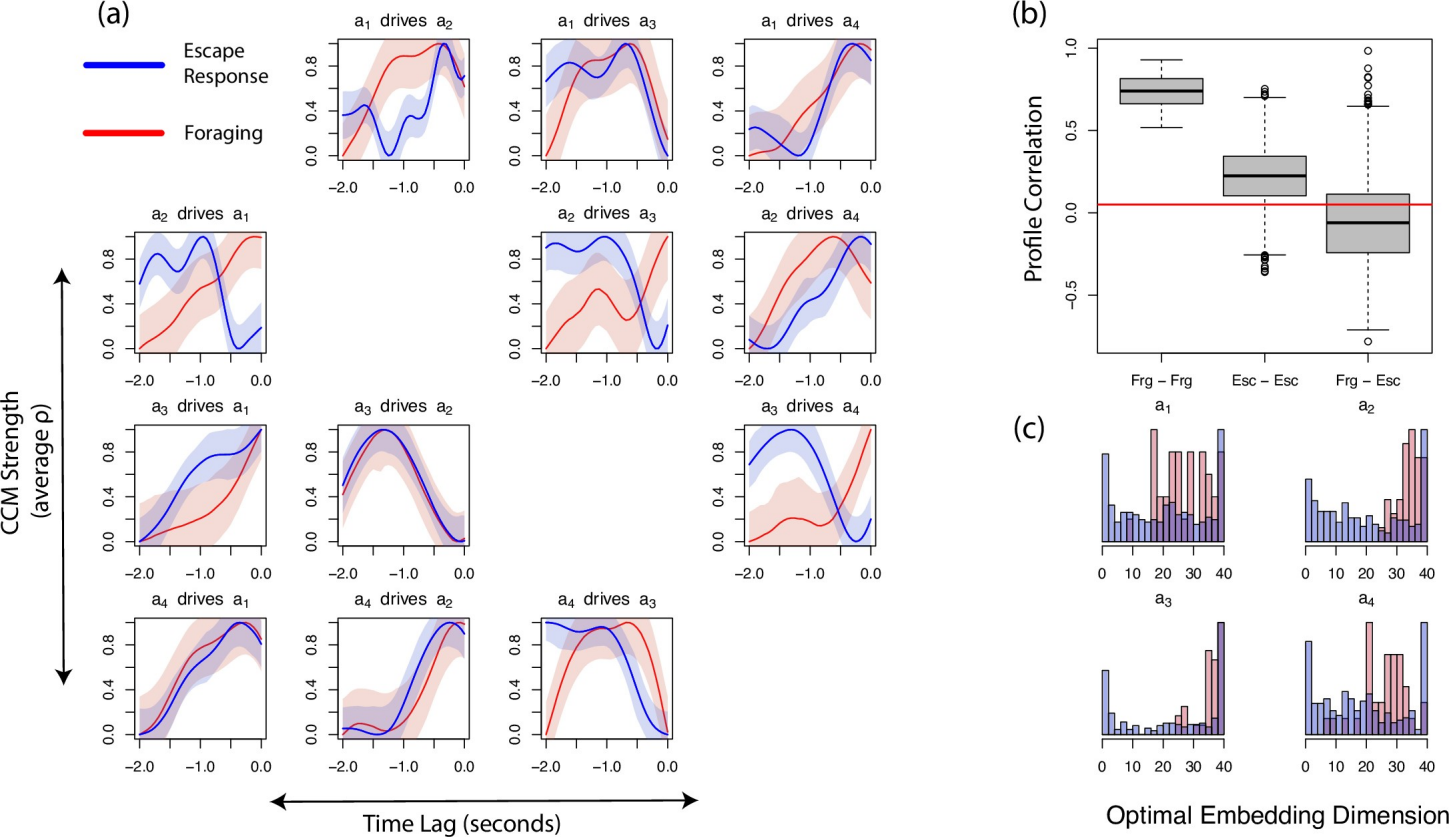
Comparing dynamics of the eigenmodes in worms foraging and exhibiting an escape response. (A) The average CCM values between the first four eigenmodes of worm position plotted against *tp* for foraging (red) and escape response (blue) worms. Shaded regions represent one standard deviation on either side of the mean. (B) Boxplots showing the correlation in dynamics between all pairs of worms. Each pair falls into one of three categories: two foraging worms (Frg—Frg), two escape response worms (Esc—Esc), or one foraging and one escape response (Frg—Esc). The red line represents the correlation between surrogate profiles (see Methods). (C) The optimal embedding dimension[s] to resolve driving dynamics in foraging (red) and escape response (blue) worms.

Looking first at N2 wildtype worms during foraging (data from [15]), we find a high degree of consistency in the shape interaction profiles across individual worms (Fig 2). However, the profiles shown here are normalized between 0 and 1 because the magnitude of CCM strength (measured by predictive accuracy) varies between worms (see S1 Fig). These profiles indicate that there exist characteristic timescales of interaction specific to particular eigenmode pairs that remain consistent throughout the foraging behavior. Indeed, contrasting these foraging interaction profiles with those obtained from the same type of worm during an escape response elicited by an aversive stimulus (data also from [15]), shows that the interaction profiles are not only consistent, but also specific: a clear qualitative difference in shape is evident in each profile between the foraging and escaping worms (Fig 3A).

We measure the similarity in worm’s profiles by calculating the mean correlation between their eigenmode interaction profiles. Calculating the average correlation between all pairs of individuals (12 foraging and 91 escaping) shows that the average correlation between pairs of foraging worms is greater than that between pairs of escaping worms (t-test *p* < 10^−6^), implying that there is more variability in escape response behaviors than in overall foraging behavior (Fig 3B). Furthermore, i) pairs of worms exhibiting the same category of behavior have a higher correlation than those exhibiting different categories of behavior (Fig 3B); and ii) the average correlation between categories of behavior is not significantly different than those between random surrogate data (Fig 3B; see Methods). Taken together, this suggests that the interaction profiles provide a quantitative characterization of these complex suites (categories) of behavior that is consistent and specific at the individual category level, and distinctive and meaningful in between-category comparisons.

The interaction profiles so far discussed were generated using 10-dimensional embeddings (see Methods), but we find that the structural consistency of these profiles is also robust to embedding dimension, across a very broad range (S2 Fig). Despite this robustness to embedding dimension, examining the effect of embedding dimension on the strength of interaction independently may still yield additional insight into dynamical differences between behaviors. By keeping the time delay for each ordered pair of eigenmodes fixed, we examine which embedding dimensions reveal the strongest causal interaction (see Methods). Interestingly, we find that the average optimal embedding dimension for eigenmode dynamics is significantly lower (t-test *p* < 10^−6^) for worms exhibiting an escape response than for those that are foraging (Fig 3C). Furthermore, for foraging individuals, the driving dynamics of *a*_1_ and *a*_4_ can typically be resolved in fewer dimensions than those of *a*_2_ and *a*_3_, which show a strong left skew (t-test *p* < 10^−6^ for all pairs except [*a*_2_, *a*_3_] and [*a*_1_, *a*_4_]).

**Fig 4:**
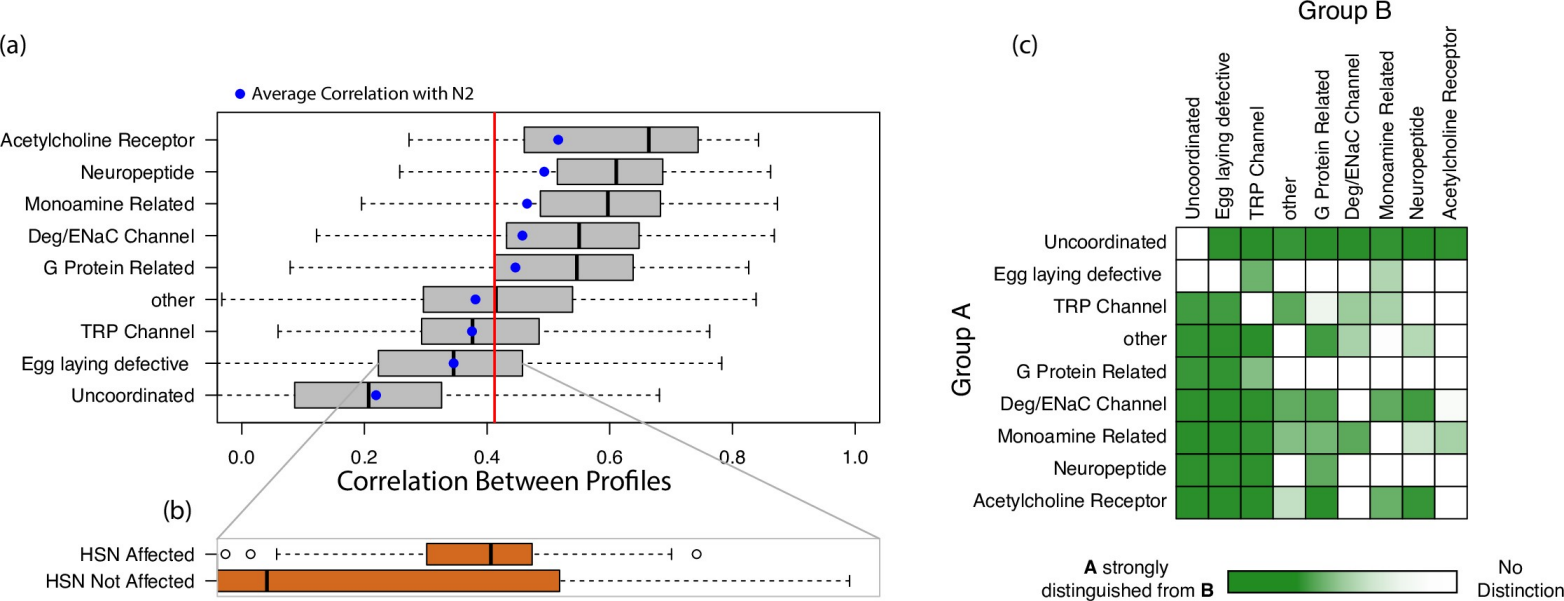
(A) Differences in dynamics between pairs of phenotypically similar mutants. The red line indicates the median correlation of all mutants from each other. The blue dots represent those strains profiles’ average correlation with that of N2 wildtype profiles. The boxplots are ordered from top to bottom in decreasing median correlation between strains, where higher correlation indicates more similar dynamics (less variance) between profiles. (C) Correlation between profiles of strains of two subgroups of “egg laying defective” strains: those that affect hermaphrodite specific motor neurons (HSNs) and those that do not. Note that this difference is not identified by [7] (S5 Fig). (C) Groups that show significantly distinct interactions profiles from each other (see Methods). Note that although some groups show lower variance, they are not necessarily discernable from each other.

A number of mutations are known to affect locomotion, and previous studies have made great strides in quantifying and categorizing the locomotory phenotypes caused by thousands of mutations [7,11,12]. From recordings of over 6,000 individuals encompassing 287 distinct mutations, we generated average interaction profiles (see Methods) for each mutation and calculated average correlation between all pairs of mutations. Each mutation falls into one of nine categories of “phenotypically or functionally similar” as defined in [7]. Calculating the average correlation between distinct mutant strains’ profiles showed that phenotypically similar mutations had significantly higher correlation in their profiles than the average across all strains for 5 out of the 9 categories (t-test *p* < 10^−6^ for all 5). The “egg laying defective” and “uncoordinated” groups showed average within-group correlations that were significantly less than the average difference between all mutants (t-test *p* < 10^−6^). This implies that strains within these two categories have variable and dissimilar patterns of behavior. When we split the “egg laying defective” category into mutants with hypothesized or confirmed effects on hermaphrodite specific motor neurons (HSNs), neurons essential for normal reproduction [16], and strains without known effects on HSNs (see S1 Table), we find that strains with mutations affecting HSNs exhibit more similar behavior than those that do not (t-test *p* < 10^−4^, Fig 4B). Both subgroups, however, still show relatively high variance when compared to the differences seen within other groups of mutations.

Although some groups show lower variance (higher average correlation) than others, they are not necessarily distinct from each other. To measure how distinct two groups are from each other, we identify the most similar strain (strain with the highest average correlation) to each strain in the two groups. If strains of one group tend to have a most similar strain that is also in that group, then that group is distinct from the other (see Methods). This process allows for directionality in these distinctions: group A may be distinct from group B, but group B may not be distinct from A. Mutations affecting acetylcholine receptors show the lowest variance, however profiles of most other categories of mutations (7 out of 9) cannot be distinguished from them (Fig 4C). Interestingly, profiles of acetylcholine receptor mutations are the most similar to that of N2 wildtypes (Fig 4A). Monoamine related mutations are moderately distinct from acetylcholine receptor-affecting mutations (Fig 4C). Uncoordinated individuals have the most distinguishable profiles: they are entirely distinguishable from every other group of mutation, and every other group can be distinguished from them. Performing this analysis on the *egl* mutation subgroups (affecting/not affecting HSNs) shows that the two groups are indeed distinct from each other (see Methods).

## Discussion

Interaction profiles measure the coupling strength between variables as a function of time. Here, we use CCM as our measure of coupling strength [9]. CCM can be thought of as measuring how consistently specific patterns in one timeseries are followed by specific patterns in another, and if so, with what delay. This approach follows that of [7], in which they measured the frequency of specific patterns in the eigenmode timeseries. Here, instead of quantifying the frequencies of patterns, we are measuring how consistently specific patterns in one eigenmode timeseries are followed by patterns in another. This, in effect, measures relationship between the modes instead of characteristics about any individual timeseries.

Worms exhibiting different categories of phenotypic behavior show significantly greater differences in their interaction profiles than those of the same behavior. Within a single strain, the interaction profiles of escaping individuals show higher variation than those of foraging worms. This may be due to slight differences in the stimuli triggering the escape response (e.g., exactly where on the worm the stimulus hit, or the position the worm was in when the stimulus occurred) and future work could explore the exact drivers of escape response behavior. Still, in accordance with previous findings [6,17,18] the level of correlation seen in escape response profiles is higher than that of our null expectation based on a surrogate test (see Methods), suggesting there are consistencies in escape response behaviors across individuals.

When comparing correlations in the interaction profiles between foraging and escaping individuals, we find that individuals exhibiting the same behavior have higher correlation than comparing between behaviors. In fact, the correlation between the profiles of these differing behaviors is not significantly different from that of our null surrogates’ correlation.

It is surprising that foraging worms have more correlated profiles than escape response individuals given that previous work has found that escape response is highly stereotypical [6,17,18]. However, this can be explained by the temporal nature of these profiles. Although the profiles taken as a long-term average for foraging worms are highly correlated, we find that when profiles are calculated as distinct 20-second windows there is less correlation between foraging profiles than that of escape response (S3 Fig). This suggests that for these N2 wildtypes, the general behavioral space that comprises foraging varies little from worm to worm, however, the trajectory (sequence) along which they progress through that behavioral repertoire is highly variable. Future work may explore the potential utility of quantifying interaction profiles in such short temporal segments to quantify behaviors over time.

Different classifications of mutations show different levels of variance between their component strains’ interaction profiles (Fig 4A). For example, mutations affecting acetylcholine receptors show relatively low variance in their interaction profiles while those of *egl* and *unc* mutations show high variance. Profiles of worms with mutations affecting acetylcholine receptors show the highest correlation to the profiles of N2 wildtypes (Fig 4A), suggesting that these mutations cause relatively few phenotypic changes, or cause changes that are not detected with the parametric choices used herein. The high variance in the *egl* and *unc* mutations can be attributed to the known variety of phenotypes caused by genes in multiple pathways within these two general groupings, as well as overlap between these categories (e.g. [19,20]). Although these two groups do not seem to describe one specific set of dynamics, there may be patterns among specific strains within these groups. For example, *egl* mutants with known effects on HSNs [16,21–25] have less variation in their profiles than the rest of the *egl* group on average (Fig 4B). Although there is less variance in interaction profiles among *egl* mutations with known effects on HSNs than those without, there is still relatively high variance in both groups. In addition to controlling vulval and uterine muscles, HSNs are known to help regulate feeding [26] and some HSN mutations cause uncoordinated locomotion [27]. Furthermore, the mutations classified as having a potential effect on HSNs disrupt a variety of functions, ranging from HSN cell migration in development to G-protein coupled receptors expressed in HSNs (e.g. [16,22,28]), and can play roles in the function of additional neuron types and pathways (e.g. [23]). The multiple roles of HSNs and of mutations classified as affecting HSNs could explain some of the variability among the worms in this group.

Mutations with effects on acetylcholine receptors showed the lowest variance in their interaction profiles; however, most other strains cannot be distinguished from them (Fig 4C). This can be imagined graphically as two clusters of points with one cluster tightly grouped within the other. If a point lies outside the inner cluster, it can be confidently determined that it is not a member of the inner cluster. However, if a point lies within the inner cluster, it cannot be confidently distinguished from the outer cluster. This may imply that the dynamics shown in acetylcholine receptor affecting groups are somewhat representative of the overall average dynamics seen in all strains. In fact, they show the most correlated profiles with the N2 wildtype profiles. This result was not seen in all groups with low variance. For example, the interaction profiles of strains affecting Deg/ENaC channels are largely distinguished both from other groups and other groups from them. Analogously, this can be graphically imagined as two non-overlapping clusters of points. It is possible that mutations affecting Deg/ENaC channels cause distinct changes in worm movement which manitfest here as a distinct difference in their interaction profiles.

Our results resonate with that of Brown et al. [7], who used a nonparametric approach to cluster strains into phenotypic groups based on similarities in frequencies of repeated positions. The two methods broadly agree (S4 Fig), however, interaction profiles can show greater sensitivity in making some distinctions. We find that both methods identify high variance in the *unc* group and low variance among individuals with mutations affecting acetylcholine receptors. Also, both methods find that although individuals with mutations affecting acetylcholine receptors show low phenotypic variance, other groups cannot be confidently distinguished from them (i.e. they cluster within other groups).

However, there are some groups that were found to be distinct only with one method and not the other. For example, mutations affecting neuropeptides were distinct from those affecting G-protein coupled receptors based on their interaction profiles (Fig 4C) but were not distinct from each other in [7]. Further, the differences identified here in HSN-affecting strains is not identified in Brown et al. (S5 Fig). Still, there are some differences identified in [7] that are missed with the interaction profiles described here. For example, Brown et al. found that monoamine related mutations are phenotypically distinct from those affecting neuropeptides while their interaction profiles were not significantly different (Fig 4C). It is possible that some dynamics identified by Brown et al. are not resolved with the methods described here. For example, certain behaviors (motifs) identified in their work may be best resolved in lower-dimensional spaces, as seen when comparing escape response dimensionality to that of foraging (Fig 3C). Interaction profiles here strictly measure how well dynamics are resolved in 10-dimensions with a 1-timestep timelag, and consequently as currently and rigidly posed (adopted to avoid over-fitting), they may not optimally resolve lower (or higher) dimensional, or lower-frequency dynamics. Setting the time delay to 1 timestep is a minimal assumption approach for performing empirical dynamic modeling, reducing the potential for overfitting. It is likely that other relationships may be better resolved with flexible parametric choices. It is encouraging that even with a fixed embedding dimension, interaction profiles can identify novel phenotypic differences between strains. Future work should explore the potential of exploring interaction profiles without fixed parameters.

Interestingly, when examining the effect of dimensionality on foraging and escaping N2 worm behavior representation, the dynamics of *a*_2_ and *a*_3_ show lower optimal embedding dimensions for escape than for foraging. We note that reduction in dimensionality of dynamics is also observed in other systems under atypical or stressful conditions, such as in brain activity preceding an epileptic seizure [29].

Beyond exploring interaction profiles in other embedding dimensions, it would be interesting to consider other metrics to compare similarities between these profiles. Perhaps certain relationships (different panels in Figs 2 and 3A) are more indicative of certain changes in worm behavior. The approach used here is minimalistic in that it makes very few assumptions and is consequently and intentionally far from optimized. It is possible that differences between distinct strains may become further resolved by a more exhaustive exploration of parameters involving different eigenmodes, different distance metrics, or different embedding dimensions etc.

Future work may explore other causality criteria to generate interaction profiles (e.g., [5,30]). CCM is specifically defined for dynamic systems where there is weak to moderate nonlinear coupling and no synchrony [9]. Thus, it would be interesting to consider the extent to which synchrony might influence these eigenmode relationships (e.g., [31]).

Nonetheless, we find interaction profiles can readily reveal novel distinctions between groups of mutations without exhaustively testing parameters (distance metric, embedding dimension, eigenmodes tested, etc.). These findings shed light on the potential of using these complex relationships between eigenmodes as a classifier of worm behavior. Future work should explore the extent to which further distinctions between strains can be made with different parametric considerations.

## Conclusion

This work follows that of Brown et al. [7], who showed that that specific patterns can be found within individual eigenworm timeseries to characterize worm behavior. Here, we show that patterns for characterizing worm behavior exist beyond individual eigenworm timeseries. Namely, we find that time-delayed causal relationships (measured with CCM [9]) also exist between eigenworm timeseries. The profiles generated by these delayed interactions between eigenmodes provide a versatile metric for measuring the similarity between worm behaviors. These profiles offer a flexible way to compare transient behaviors, such as escape responses, to that of longer-term behaviors, such as foraging, while also identifying subtle variations in behaviors attributed to genetic mutations that were not previously detectable.

## Materials and methods

### Data

We utilized the eigenmode time series of 12 foraging worms and 96 (91 post-processing) worms exhibiting an escape response from [32]. Five escaping worms were left out of our analysis as done in [32] since these worms hardly moved. We also used the eigenmode time series of over 9,000 individuals from [8] available on http://openworm.org/, https://www2.mrc-lmb.cam.ac.uk/groups/wschafer/WormBehaviorDatabase.tmp.html and https://zenodo.org/communities/open-worm-movement-database/. The mutant categories were classified by [7].

All code and processed data used to generate figures and results is publicly available at https://github.com/SugiharaLab/Projects/.

### Methods

To analyze the dynamics of 12 foraging worms [32], we performed convergent cross-mapping (CCM [9]) between all pairs of the first four eigenmodes. To do this, 200 time indices (*t*) were randomly selected from the time series (of 33,600 values) such that none were blank (NA or NaN) and used as our library in CCM [9]. We embedded these 200 values of *t* in ten-dimensions (E = 10), taking time lags to make points in the form [*x*_*t*_, *x*_*t-1*_, *… x*_*t-9*_]. This embedding was then used to make predictions on the target time series (at time *t—tp*) as described in [9]. We repeated this 250 times, selecting a different sample of 200 time indices each time, and the average correlation coefficient between observed and predicted values was used as the resolved CCM value for the time delay (*tp*) tested. This was repeated for *tp* values from -32 to 0 (equivalent to -2 to 0 seconds) to quantify how the strength of the interactions change with different delays (see [4]). This process was repeated for all pairs of the first 4 eigenmodes (4x3 = 12 total) across all foraging individuals (12), creating 12 plots of *tp* vs. CCM for each pair for each of the 12 individuals (Fig 2). The average interaction profile (red lines Figs 2 and 3A) is the normalized average across all individuals for each pair of eigenmodes.

To calculate the optimal embedding dimensions (E) for each individual, a similar process was done, however *tp* was set to the value that gave the highest CCM value in the average interaction profile for the pair of eigenmodes tested with ten dimensions. E (number of lags in the embedding) was tested from 2 to 40 with the optimal E being the number of lags that produced the highest CCM value.

For the 91 escaping worms tested [32], time series were 600 values long and sampled at 20 Hz with a laser pulse to the head occurring at 10 seconds (200th time index). Because we are only interested in escape response behavior, the first 200 values are removed from each time series, making the remaining time series 400 values long—much shorter than the 33,600 values of foraging behavior. The analysis follows similarly as that of the foraging analysis; however, because the timeseries were much shorter, we were able to compute CCM on the entire timeseries without needing to take random samples. Also, an “exclusion radius” of 10 values was also implemented, making nearest neighbors at least 10 indices (half a second) apart when making each prediction (see [33]). *tp* values were then tested from -40 to 0 (still equivalent to -2 to 0 seconds due to sampling frequency) to make the interaction profile for each escape response individual.

To calculate the average correlation between two individuals’ profiles, we calculated the Spearman correlation between all pairs of eigenmode profiles. We then summarized these 12 values by taking their mean.

Because the two datasets were sampled at different frequencies, values of the foraging dataset were interpolated in order to compare the difference between foraging and escape response individuals (S6 Fig). This allows for values to line up in time between foraging and escaping interaction profiles. To measure the significance of these correlations, we compared these values to that of random surrogates. Surrogate profiles were generated by shuffling the phases of a Fourier transform of the eigenmode timeseries, thus preserving the power spectra within the surrogate shuffles. We then performed CCM to generate interaction profiles for these surrogates.

Mutant strain data was downloaded from the Worm Behavior Database from the Schafer Lab: https://zenodo.org/communities/open-worm-movement-database/. Time series were filtered to only include those which had less than 25% NAs in the data and over 200 indices. This left 6,376 individuals encompassing 287 distinct strains. The analysis to generate interaction profiles for individuals of specific strains follows analogously from that of the foraging worms. However, because this data set was sampled at 30 Hz, *tp* was set to -60 to 0, only testing every sixth value to reduce computation time. To compare the correlations between each strain, the interaction profiles for genetically identical individuals were averaged and correlations between strains were taken between the averaged interaction profiles. This gave us a 287 x 287 symmetric correlation matrix of the average correlation between the profiles of all pairs of strains. These values were then used to generate the boxplots shown in Fig 4A and 4B by considering the average correlations between strains within specific categories defined by [7].

This correlation matrix was also used to test whether the groups were distinct (Fig 4C). To measure whether two groups were distinct from each other, we considered every pairwise combination between two groups (group A and group B, Fig 4C). For each strain in group A, we considered which other strain had the most similar interaction profile (highest correlation) out of all other strains in group A and B. After doing this for each strain in group A, we calculated the percentage of strains that had their most similar strain also in group A. This percentage was then recalculated 1000 times, however, the associations of which group each strain belonged to (A or B) was randomized each time. The level of distinction was measured as the fraction of randomized percentages that were less than that of the non-randomized percentage. In Fig 4C, the lowest level of significant distinction (lightest shade of green) corresponds to 80% of the randomized data showing less distinction than the real data and the darkest shade corresponds to 100%.

## Supporting information

S1 Table Strains that were identified to have an effect on hermaphrodite-specific neurons (HSNs) and those that did not have a known effect on HSNs. (DOCX)

S1 Video Each eigenmode can be represented as a node within a dynamical system, where the value of a node at time *t* is driven by causal influences of the other nodes. These causal influences (dynamics) can occur with a delay such that the value of a current node has a lagged effect on other nodes. The arrows represent direction of causal influence with darker arrows representing a stronger causal influence. Each frame of the video represents a time-delay between 2 and 0 seconds. For example, the first frame (delay = 2 seconds) represents how the values of the nodes will influences each other in 2 seconds. However, note that the only relationship that is relatively strong with this large of a delay is the effect of *a*_3_ on *a*_2_. The complexity seen in *C. elegans* behavior can (at least partially) be attributed to eigenmode relationships occurring at varying timescales; at any given time, each pair of eigenmodes may be in a different “phase” of their relationship allowing for many possible combinations of active causal influences (sets of opaque arrows existing at the same time).
(MP4)

S1 Fig Timeseries may have different levels of predictability. This causes resolved CCM values to change depending on the predictability of the target time series. (A) shows the cross mapping skill (correlation between observed and predicted values) between a1 and a2 over time for a foraging worm. Note that predictability varies over time. (B) shows the unnormalized profiles for the 12 foraging worms (grey) and their average (red). Note that even without normalizing, the average profiles maintain approximately the same shape. This can be explained by the fact that although the different individuals may have varying levels of predictability in their respective timeseries, the shapes of these profiles are relatively consistent.
(TIF)

S2 Fig The averaging interaction profiles for 12 foraging worms calculated with varying embedding dimensions. Interaction profiles change smoothly depending on choice of embedding dimension: dynamics resolved in five dimensions may be different than those resolved in twenty.
(TIF)

S3 Fig Comparing the profiles of escape response and foraging behaviors, both in 20-second windows. To generate the 20-second foraging profiles, we chose 25 non-overlapping 20-second windows from each of the 12 foraging individuals, and calculated 300 (25x12) interaction profiles. We then measured the correlations between these profiles. The profiles of escape response are greater than that of foraging to a statistically significant level (p<10^−6^).
(TIF)

S4 Fig Plotting the Mahalanobis Distance between mutant strains from [7] versus the correlation between respective interaction profiles reveals the two measurements correlate significantly (p-value < 10^−6^), however this correlation is relatively weak (Pearson’s ρ = -0.26). This analysis only considers distances of strains that clustered together in [7]. Note that some strains show similar dynamics in [7] but have different interaction profiles, however little-to-no strains show similar interaction profiles but different dynamics in [7]. This implies that interaction profiles can show higher sensitivity in making some distinctions.
(TIF)

S5 Fig Differences calculated in [7] between strains of two subgroups of “egg laying defective” strains. Those that affect hermaphrodite specific motor neurons (HSNs) and those that do not. Note, there is no significant difference between the two groups (p > 0.6).
(TIF)

S6 Fig CCM profiles for escaping and foraging worms are sampled at different frequencies (20 Hz vs. 16 Hz respectively). To account for this when finding the difference between the two, data in the foraging individuals’ time series is interpolated (red crosses) such that there is a point every 0.1 seconds of tp. This is done by calculating the value along the line between two consecutive CCM values (red circles) surrounding each 0.1 interval. This process was also repeated for the escape response behavior, however* since this sequence of values already has a value every 0.1 *tp* (every other value), the interpolated values line up exactly with resolved values. The sum of the differences (sum of the lengths of the black lines) can be calculated by taking the absolute difference between the interpolated foraging values and their corresponding escape response value.
(TIF)
