# Supplementary material for: Networks of Causal Linkage Between Eigenmodes Characterize Behavioral Dynamics of Caenorhabditis elegans
Source: PLoS Comput Biol. 2021 Sep 10;17(9):e1009329. doi: 10.1371/journal.pcbi.1009329 (PMC8494368; doi:10.1371/journal.pcbi.1009329)
Supplement: S1 Table — (DOCX) [file pcbi.1009329.s001.docx]

| **HSN Effected** | **HSN Not Effected** |
| --- | --- |
| MT1082 | MT1205 |
| MT8504 | MT1083 |
| MT1079 | MT1216 |
| CB1313 | MT1217 |
| JR2370 | MT1232 |
| MT6129 | MT1078 |
| CF263 | MT1179 |
| KP2018 | MT1444 |
| MT1241 | MT1231 |
| KS99 | MT1202 |
| MT2068 | MT1200 |
| MT2246 | CE1047 |
| MT2247 | AQ916 |
| MT2316 | MT1067 |
| MT2248 | MT155 |
| MT2293 | MT151 |
| MT1081 | MT1540 |
| MT1222 | MT1542 |
|  | MT1236 |
|  | AQ2316 |

Supplemental Table. Strains that were identified to have an effect on hermaphrodite-specific neurons (HSNs) and those that did not have a known effect on HSNs
