# Supplementary figures and images for: Networks of Causal Linkage Between Eigenmodes Characterize Behavioral Dynamics of Caenorhabditis elegans
Source: PLoS Comput Biol. 2021 Sep 10;17(9):e1009329. doi: 10.1371/journal.pcbi.1009329 (PMC8494368; doi:10.1371/journal.pcbi.1009329)

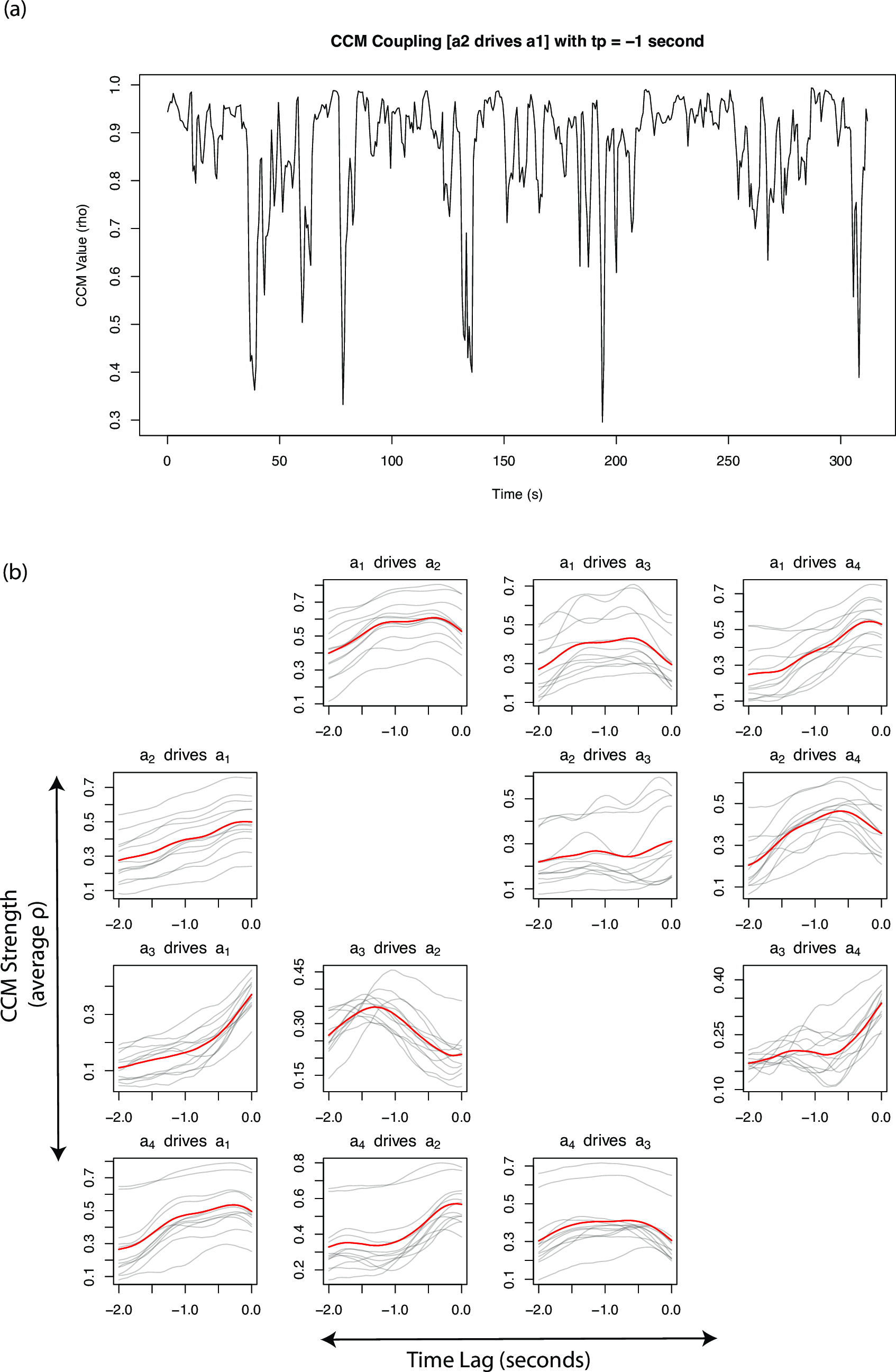

Supplement: S1 Fig — This causes resolved CCM values to change depending on the predictability of the target time series. (A) shows the cross mapping skill (correlation between observed and predicted values) between a1 and a2 over time for a foraging worm. Note that predictability varies over time. (B) shows the unnormalized profiles for the 12 foraging worms (grey) and their average (red). Note that even without normalizing, the average profiles maintain approximately the same shape. This can be explained by the fact that although the different individuals may have varying levels of predictability in their respective timeseries, the shapes of these profiles are relatively consistent. (TIF) [file pcbi.1009329.s003.tif]

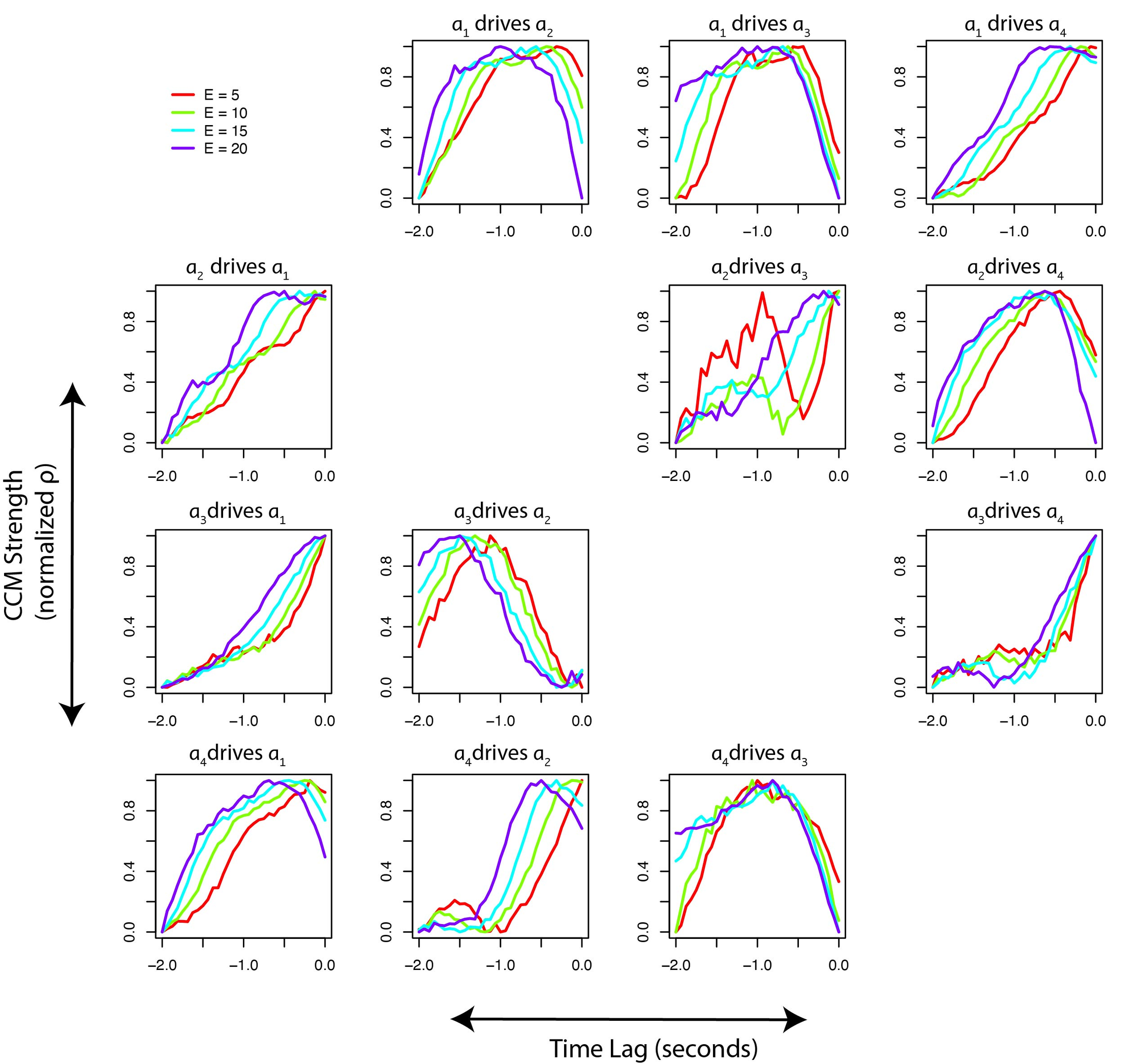

Supplement: S2 Fig — Interaction profiles change smoothly depending on choice of embedding dimension: dynamics resolved in five dimensions may be different than those resolved in twenty. (TIF) [file pcbi.1009329.s004.tif]

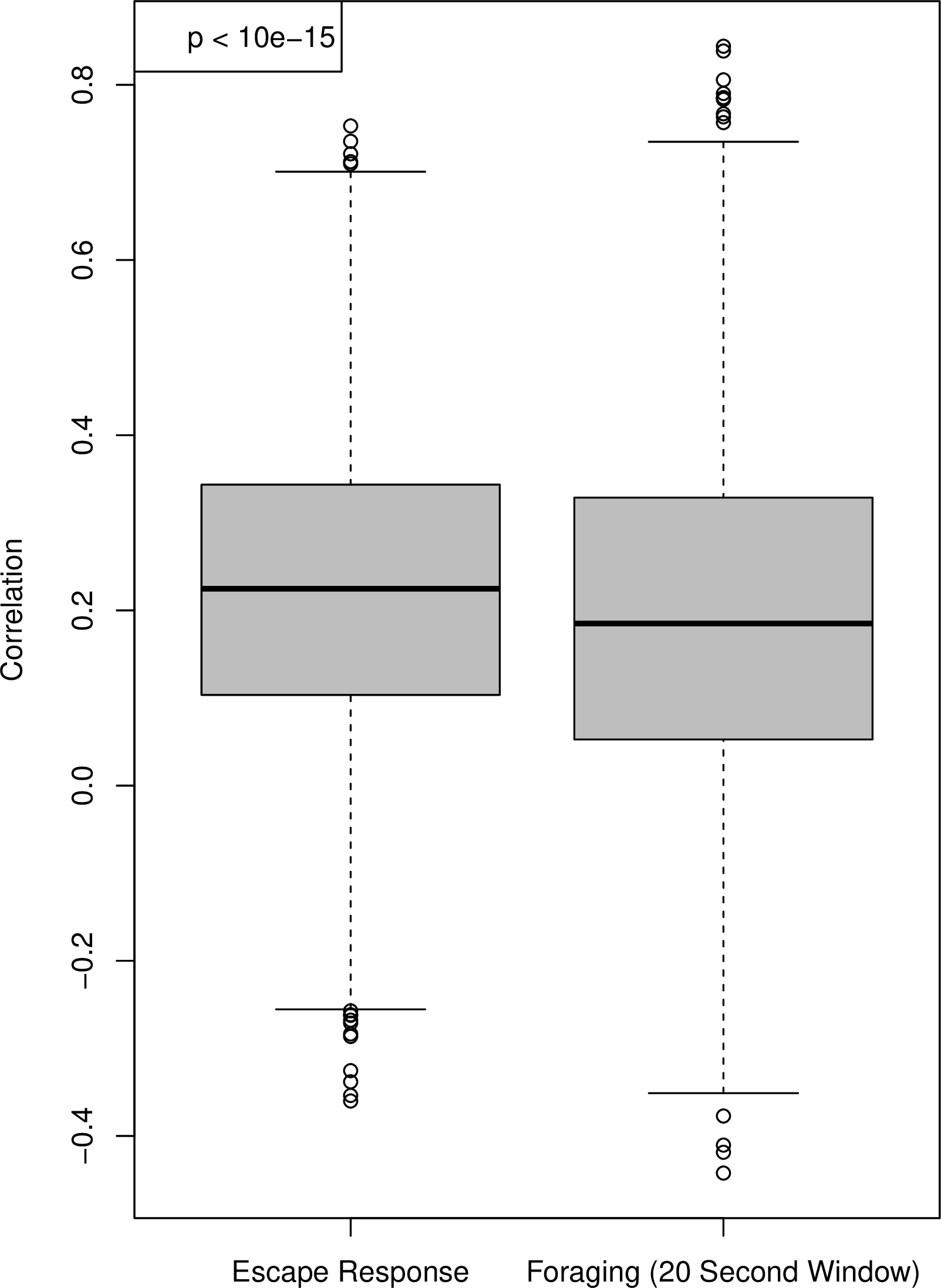

Supplement: S3 Fig — To generate the 20-second foraging profiles, we chose 25 non-overlapping 20-second windows from each of the 12 foraging individuals, and calculated 300 (25x12) interaction profiles. We then measured the correlations between these profiles. The profiles of escape response are greater than that of foraging to a statistically significant level (p<10−6). (TIF) [file pcbi.1009329.s005.tif]

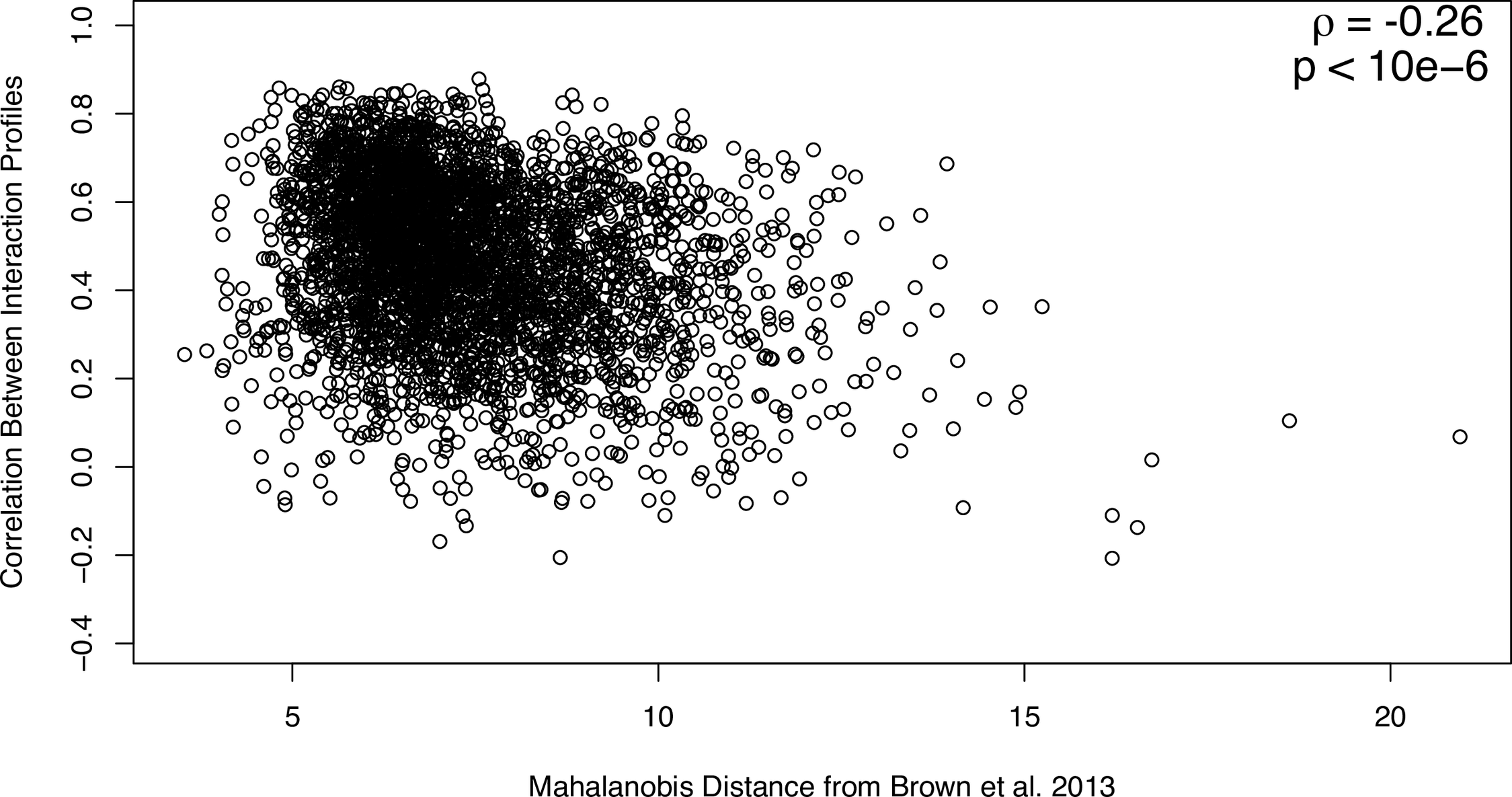

Supplement: S4 Fig — This analysis only considers distances of strains that clustered together in [7]. Note that some strains show similar dynamics in [7] but have different interaction profiles, however little-to-no strains show similar interaction profiles but different dynamics in [7]. This implies that interaction profiles can show higher sensitivity in making some distinctions. (TIF) [file pcbi.1009329.s006.tif]

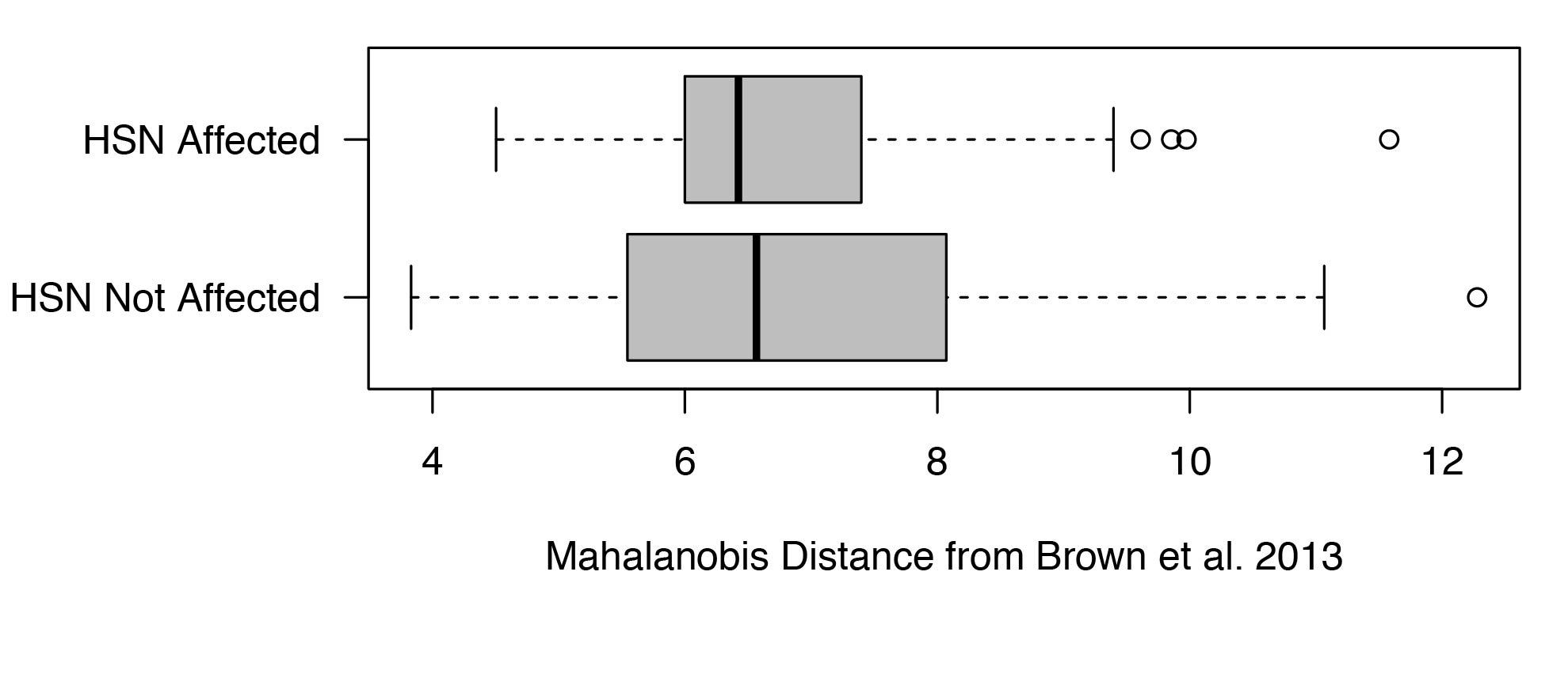

Supplement: S5 Fig — Those that affect hermaphrodite specific motor neurons (HSNs) and those that do not. Note, there is no significant difference between the two groups (p > 0.6). (TIF) [file pcbi.1009329.s007.tif]

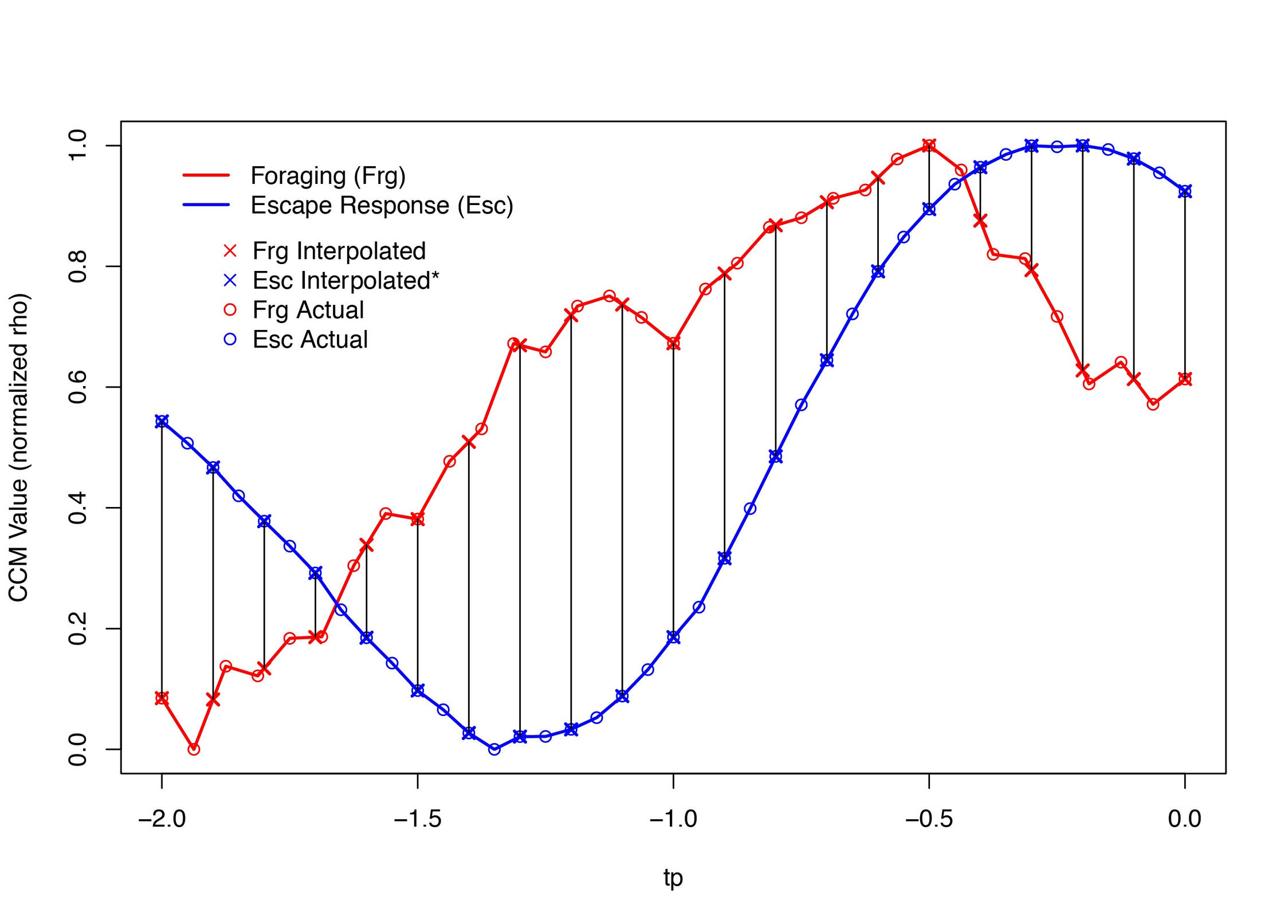

Supplement: S6 Fig — To account for this when finding the difference between the two, data in the foraging individuals’ time series is interpolated (red crosses) such that there is a point every 0.1 seconds of tp. This is done by calculating the value along the line between two consecutive CCM values (red circles) surrounding each 0.1 interval. This process was also repeated for the escape response behavior, however* since this sequence of values already has a value every 0.1 tp (every other value), the interpolated values line up exactly with resolved values. The sum of the differences (sum of the lengths of the black lines) can be calculated by taking the absolute difference between the interpolated foraging values and their corresponding escape response value. (TIF) [file pcbi.1009329.s008.tif]
